# Primed to persevere: Hypoxia regulation from epigenome to protein accumulation in plants

**DOI:** 10.1093/plphys/kiae584

**Published:** 2024-10-31

**Authors:** Daniel J Gibbs, Frederica L Theodoulou, Julia Bailey-Serres

**Affiliations:** School of Biosciences, University of Birmingham, Birmingham B15 2TT, UK; Plant Sciences and the Bioeconomy, Rothamsted Research, Harpenden AL5 2JQ, UK; Center for Plant Cell Biology, Department of Botany and Plant Sciences, University of California, Riverside, Riverside, CA 92521, USA; Plant Stress Resilience, Institute of Environmental Biology, Utrecht University, 3584CH Utrecht, the Netherlands

## Abstract

Plant cells regularly encounter hypoxia (low-oxygen conditions) as part of normal growth and development, or in response to environmental stresses such as flooding. In recent years, our understanding of the multi-layered control of hypoxia-responsive gene expression has greatly increased. In this Update, we take a broad look at the epigenetic, transcriptional, translational, and post-translational mechanisms that regulate responses to low-oxygen levels. We highlight how a network of post-translational modifications (including phosphorylation), secondary messengers, transcriptional cascades, and retrograde signals from the mitochondria and endoplasmic reticulum (ER) feed into the control of transcription factor activity and hypoxia-responsive gene transcription. We discuss epigenetic mechanisms regulating the response to reduced oxygen availability, through focussing on active and repressive chromatin modifications and DNA methylation. We also describe current knowledge of the co- and post-transcriptional mechanisms that tightly regulate mRNA translation to coordinate effective gene expression under hypoxia. Finally, we present a series of outstanding questions in the field and consider how new insights into the molecular workings of the hypoxia-triggered regulatory hierarchy could pave the way for developing flood-resilient crops.

**ADVANCES BOX**

- Gene regulatory responses to hypoxia are orchestrated across epigenetic, transcriptional, translational, and post-translational scales.
- ERFVIIs are major transducers of hypoxia via the N-degron pathway of proteolysis. ERFVII expression, localization, and function are further regulated by diverse protein kinases, transcriptional cascades, and retrograde signals from the mitochondria and ER, providing multiple points for signal integration.
- Chromatin remodeling through ERFVII-dependent enzyme recruitment and O_2_-regulated polycomb components provides an additional layer of epigenetic control that may contribute to longer-term hypoxia responses.
- Nuclear and cytoplasmic mRNA control—including alternative splicing and polyadenylation, modulated translation, and protection from degradation—is tuned to cellular signaling and energy management.

## Introduction

Oxygen is the third most abundant element by mass in the universe. Molecular diatomic oxygen (O_2_), the most stable form of oxygen, is critical to life on Earth. Plants grow at tropospheric O_2_ levels ranging from 20.9% (*p*O_2_ kP) at sea level to 9.5% at 6000 m above sea level (Dentant 2018), assuming a temperature of 0 °C. Within plant tissues, O_2_ levels can fall rapidly as a consequence of sudden flooding or be constitutively depressed due to low aeration or high metabolic activity, as in the center of a potato tuber or in meristematic regions, respectively (Weits et al. 2021). In some contexts, O_2_ dynamics serve as a cue, such as in the unfolding of the apical hook of the hypocotyl of a dicot seedling emerging from soil (Abbas et al. 2015). Early clues that plant cells respond to and prepare to persevere hypoxia came from the observation that a small set of proteins are synthesized in roots of abruptly submerged maize seedlings, but the mRNAs that could be isolated and translated in vitro encoded for a larger number of proteins (Sachs et al. 1980). In the ensuing decades, we have learned that the regulation of hypoxia-responsive gene expression—spanning from chromatin through to mRNA translation—entails a remarkable diversity of mechanisms. Here, we review the coordinated epigenetic and transcriptional mechanisms triggered by hypoxia and their integration with post-transcriptional and post-translational processes influenced by rapid or gradual changes in O_2_ level, mitochondrial state, or energy status. We focus on the regulation of the hypoxia responsive genes (HRGs), defined as the mRNAs that increase and are translated across cells, tissues, and species in response to rapid hypoxia (Mustroph et al. 2010; Reynoso et al. 2019), and others important for survival of hypoxic or submergence stress. These advances in understanding provide opportunities for improving the flooding resilience of crops.

### Oxygen sensing via the N-degron pathway of proteolysis

**Figure 1.**
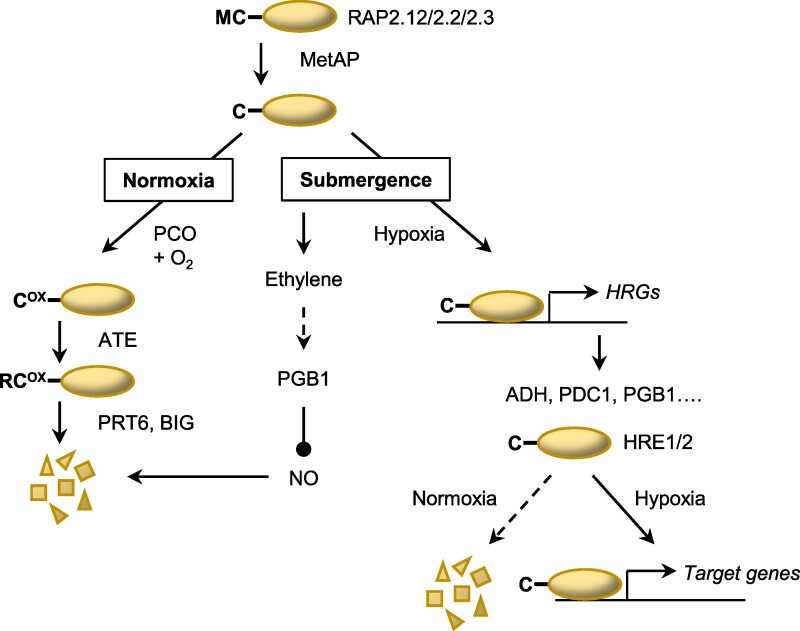
Oxygen sensing via ERFVIIs and the N-degron pathway. RAP-type ERFVII transcription factors bear a cysteine residue at position 2, which is exposed by the co-translational action of methionine aminopeptidases (MetAPs). Under aerobic conditions, Cys2 residues are oxidized by PCO enzymes. Subsequent N-terminal arginylation, catalyzed by arginyl tRNA transferase enzymes (ATEs) creates a recognition signal (N-degron) for the candidate E3 ubiquitin ligases, PROTEOLYSIS6 (PRT6) and BIG/DARK OVEREXPRESSION OF CAB1/TRANSPORT INHIBITOR RESPONSE3 (BIG), which target ERFVIIs for proteasomal degradation. NO promotes degradation through the N-degron pathway through an unknown mechanism. In hypoxic conditions, RAPs are stabilized and transcriptionally activate multiple HRGs, including *ALCOHOL DEHYDROGENASE1* (*ADH1*) and *PYRUVATE DECARBOXYLASE* (*PDC*), which encode enzymes involved in fermentation, as well as 2 other ERFVIIs: *HRE1* and *HRE2*. HRE1 and 2 are also subject to N-degron pathway-mediated degradation under normoxia. Under submergence conditions, rapid accumulation of ethylene occurs before the cellular O_2_ tension drops sufficiently to stabilize ERFVIIs. Ethylene signaling induces synthesis of PHYTOGLOBIN1 (PGB1) which scavenges NO, leading to ERFVII stabilization, priming the plant to respond to subsequent hypoxia.

Plant response to a drop in available O_2_ is coordinated by the ethylene-responsive factor Group VII (ERFVII) transcription factors, which serve as the primary activators of HRG expression (Bailey-Serres et al. 2012; Bui et al. 2015; van Dongen and Licausi 2015; Zubrycka et al. 2023). In Arabidopsis, the PLANT CYSTEINE OXIDASE (PCO) N-degron pathway connects gene expression to O_2_ availability via O_2_-dependent degradation of 3 constitutively expressed RELATED TO APETALA (RAP)-type ERFVIIs—RAP2.12, RAP2.2, and RAP2.3—and 2 hypoxia-inducible ERFVIIs: HYPOXIA RESPONSIVE ERF 1 (HRE1) and HRE2 (Gibbs et al. 2011; Licausi et al. 2011a). These share a conserved N-terminal MCGGAI(I/L)(A/S)D motif; under normoxia, PCO enzyme-catalyzed oxidation of Cys2 and subsequent arginylation creates a degron for PROTELYSIS6- and BIG/DARK OVEREXPRESSION OF CAB1/TRANSPORT INHIBITOR RESPONSE3-mediated degradation by the 26S proteasome (Gibbs et al. 2011; Licausi et al. 2011a; Weits et al. 2014; White et al. 2017; Zhang et al. 2024) (Fig. 1). Protein turnover by the N-degron pathway requires nitric oxide (NO) in addition to O_2_ (Gibbs et al. 2014), and accumulation of ethylene under submergence acts as an early signal for hypoxia by augmenting ERFVII stabilization through NO scavenging by PHYTOGLOBIN1 (Hartman et al. 2019). Although O_2_-dependent turnover of ERFVIIs is conserved in monocotyledonous crops (Mendiondo et al. 2016; Vicente et al. 2017; Loreti and Perata 2023), rice contains an atypical ERFVII, SUBMERGENCE1A (SUB1A), which evades degradation through masking of the N-terminal degron, contributing to its prominent role in submergence tolerance (Gibbs et al. 2011; Lin et al. 2019). The N-degron pathway not only regulates metabolic responses to hypoxia but also orchestrates O_2_ regulation of development by controlling the abundance of the transcription factor LITTLE ZIPPER 2 (ZPR2) and the Polycomb Repressive Complex 2 subunit, VERNALIZATION2 (VRN2), which also contain Cys at position 2 (Gibbs et al. 2018; Weits et al. 2019; Labandera et al. 2021).

### Multi-layered regulation of ERFVII transcription factors

**Figure 2.**
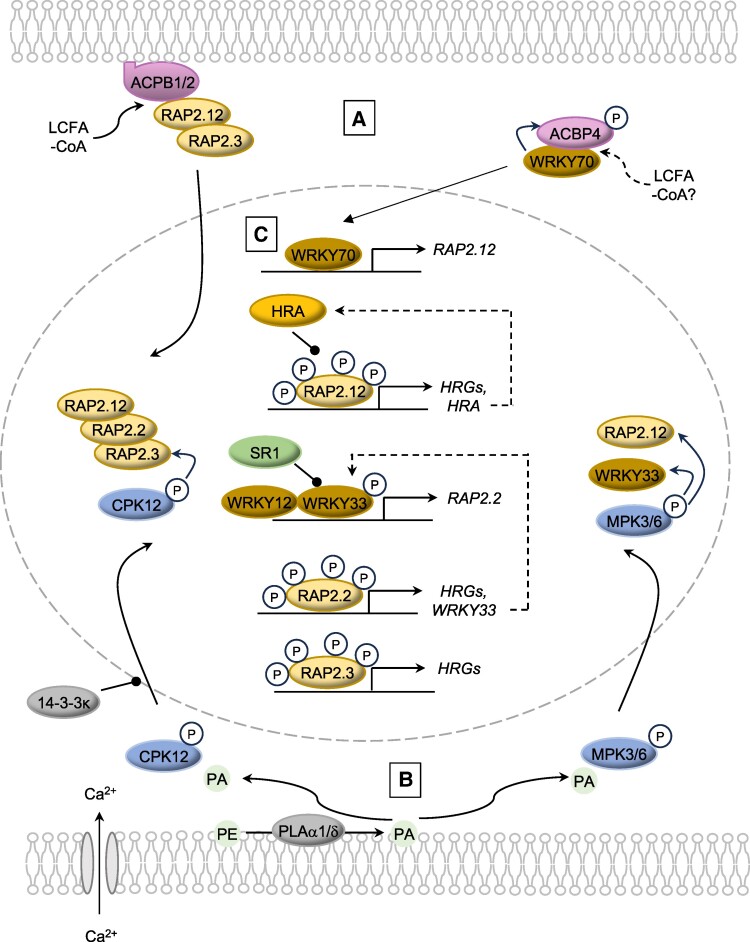
Transcriptional and post-translational regulation of ERFVII activity under hypoxia. **A)** Sequestration of transcription factors. In normoxia, RAP2.12, RAP2.3, and WRKY70 are sequestered by specific ACBPs in a state of preparedness for potential hypoxic conditions. Hypoxia stress arising from plant submergence leads to changes in the composition of the acyl-CoA pool, with long chain fatty acyl-CoAs (LCFA-CoA) triggering dissociation of transcription factors from ACBPs and facilitating their translocation to the nucleus. **B)** Pathway to phosphorylation of transcription factors. Submergence activates PLD α1 and δ, resulting in PA production from phosphatidylethanolamine (PE) in the plasma membrane. PA enhances the activity of MPK3 and MPK6 to positively regulate ERFVII-mediated signaling. CPK12 is also rapidly activated under hypoxia through Ca^2+^-dependent phosphorylation. PA promotes translocation of phosphorylated CPK12 to the nucleus where it phosphorylates ERFVIIs, increasing their stability. Nuclear translocation of CPK12 is restrained by 14-3-3κ. The activity and nuclear translocation of RAP2.12 and RAP2.2 are also regulated by TARGET OF RAPAMYCIN (not shown). **C)** Feedback regulation of transcription factors. *RAP2.12* is positively regulated by WRKY70 and negatively regulated by the HRG *HRA1*. The HRG *WRKY33* positively regulates *RAP2.2* in collaboration with WRKY12. Phosphorylation of WRKY33 promotes both its transactivation activity and its degradation by the E3 ligase SR1. Transcription factors are indicated in yellow/brown; acyl-CoA binding proteins in pink; E3 ligases in green; enzymes and transporters in gray. Arrows demonstrate positive effects and circles inhibitory effects.

Hypoxia responses must be tightly controlled, since constitutive expression of genes required for fermentative metabolism depletes reserves essential for regrowth upon reoxygenation (Licausi et al. 2011a; Cho et al. 2021). Moreover, spatial and temporal flexibility in the hypoxia response is required to accommodate local hypoxic microenvironments in tissues and organs, as well as environmental fluctuations in O_2_ availability (Weits et al. 2021; Triozzi et al. 2024). Since the discovery of the N-degron pathway as a key O_2_-sensing mechanism, it has emerged that the localization, abundance, and activity of ERFVIIs are further regulated by the interplay between sequestration, phosphorylation, and degradation, as well as transcriptional control (Fig. 2 and summarized in Table 1). These interconnected feedback mechanisms provide the capacity to tune ERFVII activity to meet the prevailing needs of the cell while providing resilience toward future challenges.

**Table 1.** Summary of ERFVII regulators, including the specific ERFVIIs they act upon and the functional relationship.

Regulator	ERFVII Target	Nature of regulation
ACBP1/2	RAP2.12 (Licausi et al. 2011a; Schmidt et al. 2018; Zhou et al. 2020) RAP2.3 (Li and Chye 2004)	Sequestration at the PM Sequestration at the PM
MPK3/6 (kinase)	RAP2.12 (Zhou et al. 2022) SUB1A-1 (Singh and Sinha 2016)	Phosphorylation Phosphorylation
CPK12 (kinase)	RAP2.12 (Fan et al. 2023) RAP2.2 (Fan et al. 2023) RAP2.3 (Fan et al. 2023) HRE1 (Fan et al. 2023) HRE2 (Fan et al. 2023)	Phosphorylation Phosphorylation Phosphorylation *In vitro* interaction only *In vitro* interaction only
TOR (kinase)	RAP2.12 (Kunkowska et al. 2023) RAP2.2 (Kunkowska et al. 2023)	Phosphorylation Phosphorylation
WRKY33/WRKY12 (transcription factor)	RAP2.2 (Tang et al. 2021)	Transcriptionally upregulated
WRKY70 (transcription factor)	RAP2.12 (Lou et al. 2022; Guo et al. 2024)	Transcriptionally upregulated
HRA1 (DNA binding protein)	RAP2.12 (Giuntoli et al. 2014)	Repression of transcriptional activity
ADA2b-GCN5 (acetyltransferase)	SUB1A-1 (Lin et al. 2023)	Enhanced transcription of *ERF66* and *ERF67* by SUB1A-1
MED25 (Mediator subunit)	RAP2.12 (Schippers et al. 2024) RAP2.2 (Schippers et al. 2024)	Transactivation of certain RAP-targeted HRGs under hypoxia
BRAHMA (SWI/SNF ATPase)	RAP2.12 (Vicente et al. 2017) RAP2.3 (Vicente et al. 2017)	Enhanced ERFVII-mediated response to abiotic stress

### Post-translational regulation of RAP-type ERFVIIs

In well-aerated (normoxic) Arabidopsis, RAP-type ERFVIIs are sequestered at the cytosolic face of the plasma membrane via interaction with 2 acyl-CoA binding proteins (ACBP1/2) but translocate to the nucleus as hypoxia ensues (Li and Chye 2004; Licausi et al. 2011a; Abbas et al. 2015; Kosmacz et al. 2015; Schmidt et al. 2018; Zhou et al. 2020). ACBP1 binds preferentially to unsaturated long-chain acyl-CoA species (18:1-, 18:2-, 18:3-CoA), which triggers dissociation of these RAPs allowing for nuclear localization (Schmidt et al. 2018; Zhou et al. 2020). It is proposed that the energy crisis imposed by hypoxia modulates the CoA pool in favor of long chain species to promote the release of RAPs and initiate the transcriptional response to hypoxia (Schmidt et al. 2018). This activates anaerobic metabolism while upregulating genes important for curtailing the response. If ATP levels drop in the absence of hypoxia, RAP2.12 is degraded by the N-degron pathway, avoiding inappropriate activation of the response. Thus, the ACBP:RAP complex is a hub that coordinates O_2_ availability and cellular energy status (Fig. 2).

RAPs are further tuned to sugar and energy availability by the central energy sensor, Target of Rapamycin (TOR), which is required for a complete hypoxia response (Kunkowska et al. 2023). Sugar activation of TOR promotes activity of RAP2.12 and RAP2.2 through phosphorylation, thus ensuring that HRG expression matches carbohydrate availability, a prerequisite for fermentative metabolism. Paradoxically, inhibiting or downregulating TOR increases nuclear localization of RAP2.12 independent of the N-degron pathway, perhaps indicating a homeostatic mechanism (Kunkowska et al. 2023). At least 2 other classes of protein kinases further modulate ERFVII activity. Mitogen-activated protein kinases (MPKs) 3, 4, and 6 are activated within 15 minutes of hypoxia and to a further extent within minutes of reoxygenation in Arabidopsis (Chang et al. 2012). Indeed, mitogen-activated protein kinase 3 (MPK3)/6 activation by submergence phosphorylates RAP2.12, resulting in increased abundance, translocation to the nucleus, and transcriptional activity (Zhou et al. 2022) (Fig. 2). Similarly, complete submergence of rice activates MPK3, shown to phosphorylate submergence tolerance-conferring SUB1A-1 at Ser161 (Singh and Sinha 2016; Lin et al. 2023). This Ser is replaced by Pro in the less effective *SUB1A*-2 protein (Xu et al. 2006). The activation of Arabidopsis MPK3/6 may be stimulated by phosphatidic acid (PA), which is liberated from membrane lipids during submergence by the action of phospholipase D (PLD) α1 and δ (Xie et al. 2015; Zhou et al. 2022). Although *pldα1* and *pldδ* mutants are hypersensitive to hypoxia, they are more tolerant of submergence, likely due to improved membrane integrity. Submergence-induced PA production is regulated via a feedback loop involving phosphorylation of PLDα1/δ by MPK3/MPK6 (Zhou et al. 2022).

A release of Ca^2+^ from intracellular stores upon hypoxia is a prerequisite for HRG activation in Arabidopsis and maize (Subbaiah et al. 1994; Sedbrook et al. 1996; Baxter-Burrell et al. 2002; Bailey-Serres and Chang 2005). In Arabidopsis, fluorescent Ca^2+^ sensors confirm a transient rise in cytosolic Ca^2+^ within 2 hours in submerged leaves (Wagner et al. 2019). Ca^2+^-dependent protein kinase 12 (CPK12) is rapidly activated under hypoxia via Ca^2+^-dependent phosphorylation of Ser186 (Fan et al. 2023). It was found that PA, which is known to facilitate nuclear import of proteins lacking a canonical nuclear localization signal (Yao et al. 2014), promotes the translocation of phosphorylated CPK12 to the nucleus where it phosphorylates RAP-type ERFVIIs, increasing their stability. Counterbalancing this is 14-3-3κ, which acts as a negative regulator of CPK12 cytosol-to-nucleus translocation (Fan et al. 2023) (Fig. 2). The question arises: why are ERFVIIs regulated by multiple distinct phosphorylation cascades? One possibility is that they help to steer ERFVII specificity in the context of physiological hypoxia. Alternatively, it may reflect the involvement of the N-degron pathway in diverse biotic and abiotic stress responses (Holdsworth et al. 2020). Given the known roles of CPK12 in salt signaling and MPKs in reactive oxygen species (ROS) and defense responses (Pitzschke et al. 2009; Chang et al. 2012; Zhang et al. 2018b), regulation of ERFVIIs by these kinases may enable integration of hypoxia responses with other environmental stress signaling pathways. A second question is: do mitochondrial signals regulate ERFVII phosphorylation, in addition to PA? This could be the case as treatment with the mitochondrial electron transport chain (mETC) inhibitor antimycin A activates Ca^2+^ release, MPK3/MPK6, and HRG mRNA accumulation in aerated seedlings (Chang et al. 2012; Zhu et al. 2023). As will be discussed, there is genetic redundancy in transcriptional activation of HRGs in response to mETC inhibition under hypoxia.

### Transcriptional regulation of RAP-type ERFVIIs

Although the Arabidopsis RAP-type ERFVIIs are constitutively expressed, they are further regulated by transcriptional feedback loops to tune hypoxia responses. ERFVII transcription is positively influenced by WRKY transcription factors, which in turn are subject to post-translational regulation. In a positive feedback loop, RAP2.2 activates *WRKY33* through a Hypoxia-Responsive Promoter *E*lement (HPRE) located proximal to the transcription start in this and many other HRGs (Gasch et al. 2016; Lee and Bailey-Serres 2019). WRKY33 then recruits WRKY12 to synergistically upregulate *RAP2.2* and amplify the hypoxia signal (Liu et al. 2021; Tang et al. 2021) (Fig. 2). WRKY33 is regulated by MPK3/6-dependent phosphorylation, which is thought to promote its transactivation activity. Phosphorylation also promotes WRKY33 degradation by the E3 ligase SUBMERGENCE RESISTANT 1 (SR1). *SR1* is predominantly expressed under O_2_-replete conditions and acts in concert with the N-degron pathway to counterbalance the hypoxia response (Liu et al. 2021), which may be important during and post stress. The role of WRKY33 appears to be specific to *RAP2.2* since transcript levels of the other 4 ERFVIIs are not correlated with *WRKY33* expression (Tang et al. 2021).

RAP2.12 is positively regulated by WRKY70 (Lou et al. 2022). In a manner analogous to the ACBP-ERFVII module, WRKY70 is sequestered in the cytosol by binding to ACBP4. Hypoxia-induced accumulation of 18:1-CoA and phosphorylation of ACBP4 promote dissociation of WRKY70 from the complex, followed by translocation to the nucleus, where it activates *RAP2.12* expression (Guo et al. 2024) (Fig. 2). WRKY70 also binds to the *ACBP4* promoter, suggesting a positive feedback loop. The kinase responsible for ACBP4 phosphorylation remains to be identified, but MPK3/6 are plausible candidates, given their role in positive regulation of the hypoxia response and their activity toward WRKY33 (Liu et al. 2021). RAP2.12 is also subjected to negative regulation by *HYPOXIA RESPONSE ATTENUATOR1* (*HRA1*), which encodes a trihelix DNA binding protein (Giuntoli et al. 2014). HRA1 interacts with RAP2.12 to curtail its activity and also negatively regulates the activation of its own promoter. *HRA1* is predominantly expressed in young shoot tissues that exhibit physiological hypoxia, thereby fine-tuning the hypoxia response during development to preserve resources for regrowth following the return to normoxia (Giuntoli et al. 2017a). Two orthologs of *HRA1* are implicated in submergence responses in rice and interact with SUB1A and the related ERFVII SUB1C in a yeast 2-hybrid assay (Jung et al. 2010).

### Epigenetic mechanisms controlling hypoxic gene transcription

While ERFVIIs are the predominant transcriptional regulators of response to hypoxia, and a mechanism connecting their activity to the perception of O_2_ deprivation is now well characterized (Gibbs et al. 2011; Licausi et al. 2011a), the presence of transcription factors alone is insufficient to accurately control or predict the signal-triggered activation of genes. Epigenetic regulatory mechanisms provide additional layers of control over gene activity. This can be directly on the DNA sequence through cytosine methylation (Zhang et al. 2018a) or via remodeling of nucleosomes—composed of histone protein octamers—that make up the chromatin scaffold supporting DNA (Bannister and Kouzarides 2011; Talbert and Henikoff 2017). The positioning of nucleosomes and the identity and post-translational modification of their composite histone variants collectively influence gene activity by impacting 3D chromatin structure, access of transcription factors to DNA binding sites, and the efficiency of RNA Polymerase II (RNAPII) recruitment and elongation (Candela-Ferre et al. 2024). Histone modifications—including acetylation, methylation, and mono-ubiquitination—occur on accessible tails of histones and, depending on their nature and location, can promote or repress transcription. These modifications are dynamic and reversible through the action of antagonistic “writers” and “erasers” but can also be mitotically stable. As such, in addition to coordinating immediate gene regulatory responses, epigenetic modifications can encode longer term memories at genes, which can be important for facilitating plant adaptation to seasonal change and recurring stresses (see Box 1).

Box 1. **Forget me not: Flooding priming and memory.**

Transient exposure of plants to stresses can induce long-term changes that promote faster or more robust responses upon stress reoccurrence (Hilker et al. 2016). These changes can be considered as beneficial “memories,” while the initial signals that trigger their induction are commonly referred to as priming events. Priming can promote short-term alterations in gene activity, protein levels and activity, or metabolite abundance, whereas longer-term changes can be induced epigenetically at “memory genes” through DNA methylation or histone modifications that persist either within or across generations (Harris et al. 2023). There is increasing evidence that plants can be primed for flooding responses. For example, wheat (Li et al. 2011), soybean (Agualongo et al. 2022), cucumber (Kęska et al. 2021), and tomato (Niu et al. 2023) all display enhanced tolerance to a second waterlogging stress following a prior nonlethal waterlogging event, and for wheat there is some indication that this can be transmitted across generations (Feng et al. 2022). For each case, stress priming was correlated with alterations in various downstream processes, such as changes in ROS levels and signaling, concomitant changes to photosynthesis, enhanced glycolysis, and the induction of ethylene biosynthesis. Despite documenting such changes, the underlying molecular perception and transduction mechanisms were not defined. Recently, a role for ethylene in establishing short-term hypoxic stress priming was reported in Arabidopsis, through promoting expression of PHYTOGLOBIN1, a potent NO-scavenger that facilitates the accumulation of ERFVII transcription factors before cellular hypoxia sets in (Gibbs et al. 2014; Hartman et al. 2019). While this mechanism preadapts plants for low-O_2_ stress during flooding, it remains to be elucidated if it can also prime plants over longer time periods. A molecular candidate for the induction of longer-term epigenetic memories of hypoxia is the PRC2 subunit VRN2 (Gibbs et al. 2018; Labandera et al. 2021). Similar to its roles in promoting a memory of winter cold through epigenetically silencing *FLC*, hypoxia-stabilized VRN2 might also promote epigenetic silencing of key loci under flooding stress to facilitate longer-term flooding resilience. As our knowledge of the epigenetic players and mechanisms controlling hypoxia-responsive gene expression and tolerance advances, it will be important to investigate their potential roles in long-term priming and the coordination of stress memory, as this could provide new solutions for enhancing stress tolerance in diverse crop species (Liu et al. 2022).

Large scale “omics” studies have revealed the global patterns of hypoxia-triggered changes to histone modifications, histone variants, chromatin accessibility, and DNA methylation. Here, we discuss current knowledge and speculate on the underlying mechanisms controlling these epigenetic responses in plants through focusing on specific enzymes and their hypoxia-responsive activities.

### Chromatin remodeling under hypoxia

#### Active chromatin

**Figure 3.**
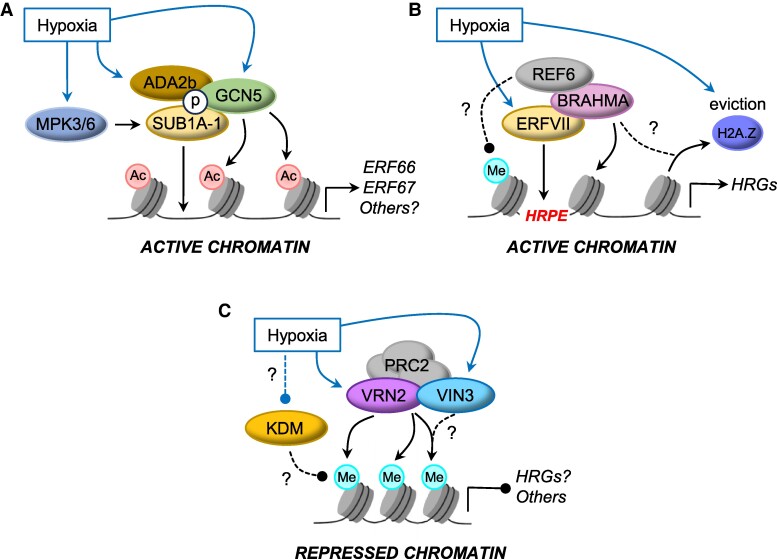
Defined and hypothetical mechanisms of chromatin remodeling under hypoxia. **A)** In submergence-tolerant rice containing the active *SUB1A-1* allele, hypoxia-triggered phosphorylation of SUB1A-1 by MPK3/6 promotes physical interaction with the ADA2b/GCN5 HAT complex. This facilitates the deposition of activating H3K9Ac (Ac) marks on SUB1A-1 target genes *ERF66* and *ERF67*, potentiating their expression under submergence. **B)** Stable Arabidopsis ERFVIIs bind to the Hypoxia Response Promoter Element and can interact with the SWI/SNF chromatin remodeler BRAHMA, reinforcing their ABA-responsive functions. BRAHMA is known to promote H2A.Z eviction, shown to occur at HRGs under O_2_ deprivation. BRAHMA interacts and colocalizes with the histone demethylase (KDM) REF6 at many loci across the genome. It is speculated that this could provide a hypoxia-responsive mechanism for histone demethylation at HRGs to further induce an active chromatin state. **C)** The PRC2 subunit VRN2 is stabilized under hypoxia and positively regulates hypoxia resilience, suggesting that it could steer H3K27me3 (Me) deposition under stress. An interactor of VRN2, VIN3, is transcriptionally induced by low O_2_ and enhances hypoxia tolerance. VIN3 may act in conjunction with VRN2 to promote a long-term repressive state at target genes. In animals, several KDMs have been defined as direct O_2_ sensors that are inhibited under hypoxia; plant KDMs may also function in a similar manner and that their hypoxia-triggered inhibition could promote retention of repressive H3K27me3 marks across the genome. Question marks and dashed lines denote hypothetical mechanisms that still require experimental validation. Arrows demonstrate positive effects, and circles inhibitory effects.

Active gene expression is associated with chromatin loosening, and HRGs display increased chromatin accessibility near their transcription start site in response to hypoxia in Arabidopsis (Lee and Bailey-Serres 2019) and submergence in rice and *Medicag*o (Reynoso et al. 2019; Reynoso et al. 2022). This indicates that chromatin relaxation is a conserved feature of low-O_2_ responses across plant species. A hallmark of gene activity is Histone H3 Lysine 9 acetylation (H3K9Ac); indeed, this mark is evident on the gene body of actively transcribing HRGs in Arabidopsis and rice (Tsuji et al. 2006; Lee and Bailey-Serres 2019). Histone acetylation is catalyzed by histone acetyltransferases (HAT), and chemical inhibition of histone deacetylase (HDAC) activity can enhance HRG expression (Tsuji et al. 2006); until recently, a mechanism connecting these enzymatic activities to hypoxia remained unknown. The rice ERFVII SUB1A-1 physically associates with the ADA2b-GCN5 acetyltransferase complex in response to its hypoxia-triggered phosphorylation by MPK3. Within this complex, GCN5 functions as a HAT for the deposition of H3K9ac. Analysis of 2 target genes of SUB1A-1, *ERF66* and *ERF67*, found their transcription is potentiated by phosphorylation-dependent recruitment of SUB1A-1 (Lin et al. 2019) (Fig. 3A). By contrast, the SUB1A-2 variant that lacks this MPK3 phosphosite fails to activate *ERF66* and *ERF67* in the presence of ADA2b-GCN5. Since SUB1A turnover is uncoupled from the O_2_-sensing N-degron pathway (Gibbs et al. 2011), this signaling cascade may help to steer its specificity under submergence, although an assessment of SUB1A-1–responsive changes to global H3K9Ac levels is lacking. Whether HAT recruitment is a more general feature of ERFVIIs is yet to be determined, but, as highlighted earlier, MPK3 in Arabidopsis phosphorylates RAP2.12 and potentiates its transcriptional activity, suggesting a potential conservation of mechanism that should be investigated (Zhou et al. 2022; Fan et al. 2023; Kunkowska et al. 2023). Alternatively, HAT recruitment by ERFVIIs in other species may not require an intermediate phosphorylation step since a majority are labile in oxygenated environments.

ERFVII association with the multi-subunit and variable Mediator Complex that connects transcription factors to RNAPII in a signal-dependent manner also appears to be important for gene activation under hypoxic stress. Interactions between RAP2.2/RAP2.12 and the MEDIATOR COMPLEX 25 (MED25) subunit promotes transactivation of a subset of HRGs in response to low O_2_ (Schippers et al. 2024). Moreover, a ubiquitin ligase controlling MED25 stability—MED25 BINDING RING-H2 PROTEIN 1 (MBR1) *–*also contributes to the regulation HRG expression, with natural variants of MBR1 differentially impacting hypoxia tolerance and adaptation of Arabidopsis to rainy environments (Castellana et al. 2024). It is not yet known if chromatin features or involvement of specific transcription factors or *cis*-elements determine which HRGs are Mediator-dependent or independent.

In addition to histone modifiers, ATP-dependent chromatin remodelers also facilitate gene activation by repositioning, ejecting, or modifying nucleosomes to increase DNA accessibility (Reyes et al. 2021). The SWI/SNF (Switch Sucrose Non-fermentable)-type ATPase BRAHMA physically associates with stabilized ERFVIIs in Arabidopsis and contributes to their ABA-related stress function (Vicente et al. 2017), indicating that BRAHMA may positively influence expression of HRGs. A recent study showed that BRAHMA colocalizes with the H2A.Z histone variant across the Arabidopsis genome (Torres and Deal 2019). H2A.Z is typically found in proximal promoter regions, and its eviction from the transcription start site region of HRGs correlates with their enhanced RNAPII engagement under hypoxia (Lee and Bailey-Serres 2019), which may be actioned via ERFVII-BRAHMA association (Fig. 3B). Arabidopsis BRAHMA can interact with and co-target many of the same genes as the histone lysine demethylase (KDM) RELATED TO EARLY FLOWERING 6 (REF6) (Li et al. 2016), which promotes an active chromatin state by reducing the levels of repressive Histone H3 Lysine trimethylation (H3K27me3; see next section). REF6 functions alongside the chromatin remodeler EIN6 ENHANCER (EEN) to repress H3K27me3 levels and promote H2A.Z eviction at *ETHYLENE INSENSITIVE 2 (EIN2*), which encodes the central regulator of the ethylene signaling pathway (Zander et al. 2019). Given the link between ethylene signaling and hypoxia responses, this concerted regulation of chromatin state at *EIN2* might help coordinate submergence responses. Although a direct connection between REF6 activity and HRGs has not been established, we speculate that hypoxia-stabilized ERFVIIs may act as docking hubs for the co-recruitment of a diverse array of chromatin modifiers—including HATs, ATP remodelers, and KDMs—at genes with a Hypoxia Response Promoter Element to synergistically activate transcription in response to low O_2_ (Fig. 3). It remains to be seen how variations in *cis*-regulating motif composition and posttranslational modification of the ERFVIIs contribute to protein interactions in this context.

#### Repressed chromatin

One of the most prevalent repressive histone modifications is H3K27me3. Although HRGs do not undergo major H3K27me3 changes in response to short-term hypoxia in Arabidopsis (Lee and Bailey-Serres 2019), it is likely that transcriptional reprogramming in response to reduced O_2_ affects the levels of this mark at certain loci, given its abundance across the genome. In mammals, H3K27me3 accumulates under hypoxia due to a reduction in the O_2_-dependent activity of 2 Jumonji-type dioxygenase KDMs that erase this mark in O_2_-replete conditions (Batie et al. 2019; Chakraborty et al. 2019). To date, a direct connection between intracellular O_2_ availability and KDM activity has not been established in plants, but related proteins have been found (Chen et al. 2011; Holdsworth and Gibbs 2020), suggesting that a similar mechanism for influencing the methylation status of chromatin under hypoxia might exist.

H3K27me3 is deposited by the conserved PRC2 holoenzyme, and flowering plants have an expanded number of genes encoding individual subunits of this polycomb complex compared with animals (Margueron and Reinberg 2011; Derkacheva and Hennig 2014). One of these, VERNALIZATION2 (VRN2), was identified as an O_2_- and NO-labile target of the N-degron pathway, suggesting that it may act as a sensor subunit that can direct PRC2 activity under hypoxia (Gibbs et al. 2018). Within hypoxic niches of the root, VRN2 has a repressive growth effect, with enhanced root system proliferation in *vrn2* mutants, while in the hypoxic shoot apex it has differential effects on flowering depending on day length and ecotype (Labandera et al. 2021). In aerial tissues, VRN2 has a repressive effect on growth, specifically through methylating histones of genes linked to cell expansion that are targeted by the PIF4 transcription factor (Osborne et al. 2024). Deposition of H3K27me3 at these loci facilitates their light-mediated repression by phytochrome B and the VRN2-PRC2 accessory protein VIL1/VRN5. As such, VRN2 may connect natural hypoxia gradients in the shoot to the control of plant growth by establishing a stable and conditionally repressed epigenetic state at key growth-associated genes. Whether this repressive growth function in shoots and roots directly influences submergence tolerance is yet to be established, but growth cessation (i.e. quiescence) is a common strategy employed by certain flood-tolerant species and ecotypes (Voesenek and Bailey-Serres 2015; Pucciariello and Perata 2024).

As well as being enriched in hypoxic developmental niches, VRN2 is stabilized in response to submergence-induced hypoxia, where it promotes waterlogging and hypoxia tolerance via unknown targets (Gibbs et al. 2018). VRN2 may contribute to short-term or transient transcriptional repression in response to low O_2_, or hypoxia-stabilized VRN2 may contribute to the induction of hypoxia-stress memory by targeting and stably repressing specific genes (see Box 1), similar to how cold-stabilized VRN2-PRC2 represses *FLC* to encode a memory of winter (Gendall et al. 2001). VRN2-PRC2 interacts with the accessory protein VIN3, a close relative of VIL1/VRN5 and a major facilitator of vernalization (Sung and Amasino 2004; Wood et al. 2006). Interestingly, *VIN3* is also induced by low-O_2_ stress and promotes hypoxia tolerance in seedlings, though not through targeting classic HRGs (Bond et al. 2009). The rate of *VIN3* induction appears to be slower than that of hypoxic-triggered VRN2 accumulation and indeed HRG induction (Bond et al. 2009; Labandera et al. 2021). The polymerization of VIN3 in association with nuclear assemblies is important for enhancing the avidity, retention, and activity of VRN2-PRC2 at *FLC* once VIN3 levels reach a critical threshold (Schulten et al. 2024). Possibly, VIN3 cooperates with VRN2-PRC2 only under conditions of sustained O_2_ deprivation to facilitate robust and long-term H3K27me3 deposition at important response genes (Fig. 3C).

### DNA methylation and hypoxia responses

Although a genome-scale evaluation of DNA methylation changes in response to hypoxia in plants is lacking, recent work deciphered a potential role for RNA-directed DNA methylation (RdDM) in survival of transient hypoxia. Loreti et al. (Loreti et al. 2019) studied ARGONAUTE1, a mediator of post-transcriptional gene silencing that regulates microRNA (miRNA) production. miRNAs influence hypoxia responses in animals, and the expression of a number of miRNAs is modulated by hypoxia and mitochondrial dysfunction in Arabidopsis and maize (Moldovan et al. 2010; Licausi et al. 2011b). Although *ago1* mutants are hypersensitive to submergence, due to effects on starch content and sugar starvation, changes to miRNA expression under hypoxia are minimal and do not correlate with changes in target mRNAs (Loreti et al. 2019). Interrogation of the submerged *ago1* transcriptome identified several mRNAs that are hyper-induced relative to wild type, including *HOMOLOG of RPW8-4* (*HR4*), which is also upregulated in lines expressing stable RAP2.12 (35S::Δ13-RAP2.12) but absent in the *erfVII* quintuple mutant (Giuntoli et al. 2017a). Considering alternative functions for AGO1, which can directly influence RNA transcription and RdDM, it was shown that *HR4* is strongly methylated in its second exon in wild type relative to 35S::Δ13-RAP2.12 and mutants of *ARGONAUTE4* (*AGO4*), a key catalytic mediator of RdDM. Correlating with this, *ago4* mutants are tolerant of hypoxic stress. This study reveals complex and still unclear involvement of post-transcriptional gene silencing and DNA methylation actioned via AGO1 and AGO4 during hypoxia and intriguingly suggests that O_2_ sensing by RAP2.12—and possibly other ERFVIIs—can influence DNA methylation on a constrained set of response genes.

### Hypoxia responses beyond the N-degron pathway

**Figure 4.**
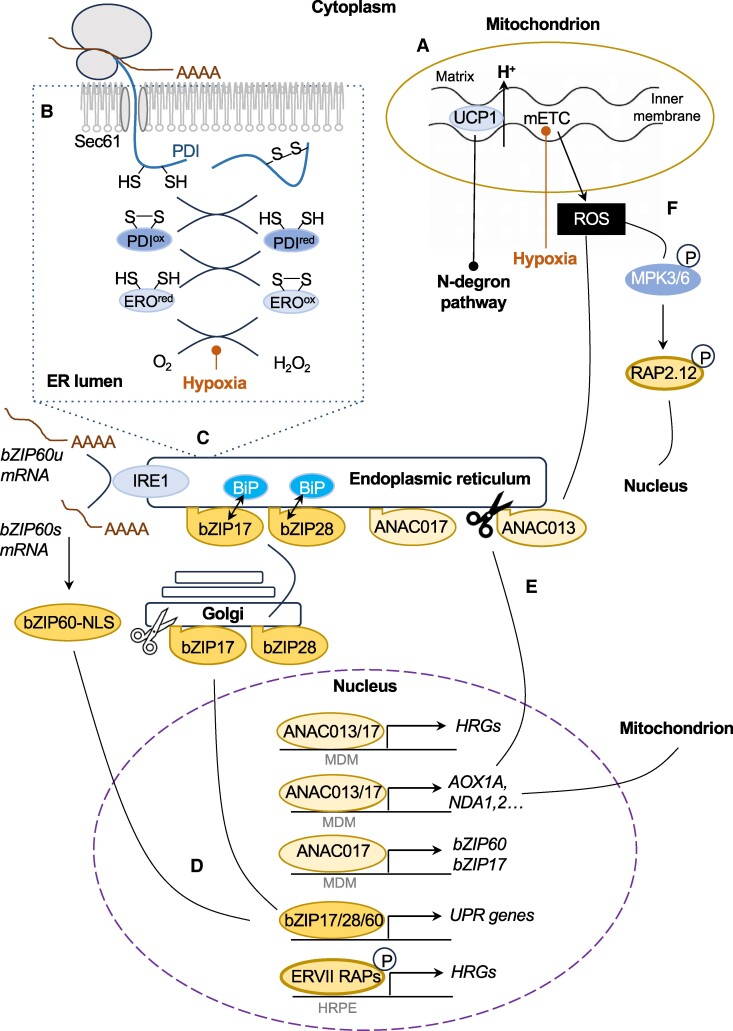
Integration of mitochondrial and ER hypoxia response. **A)** In mitochondria, low O_2_ compromises the mETC, resulting in ROS production. **B)** In the ER, hypoxia influences oxidative protein folding, depicted as a simplified scheme in the dashed box. Nascent peptides bearing thiol groups are transported into the ER via the Sec61 translocon. In the ER lumen, disulfide bonds form by the sequential action of protein disulfide isomerase (PDI) and ER oxidoreductase (ERO) enzymes, with O_2_ as the terminal electron acceptor. **C)** Protein misfolding occurs under low O_2_ and is sensed through the dissociation of heat shock protein 70 chaperones (BiPs) from the ER-tethered transcription factors bZIP17 and bZIP28. bZIP28 translocates to and is proteolytically processed in the Golgi (indicated by the white scissors icon), releasing the N-terminal portion, which translocates to the nucleus. In parallel, ER located Inositol-requiring enzyme 1 (IRE1) is activated in response to protein misfolding and catalyzes unconventional splicing of *bZIP60u* mRNA to produce *bZIP60* encoding a form of the transcription factor bearing a nuclear localization signal (NLS). **D)** Following translocation to the nucleus, the bZIP transcription factors homo- and heterodimerize to activate expression of UPR genes. **E)** Mitochondrial ROS signals release ANAC transcription factors from the ER. For example, ANAC013 is cleaved by rhomboid-like protease, RBL2 (black scissors icon) and translocates to the nucleus. ANAC013 and 017 regulate transcription of diverse genes bearing a mitochondrial dysfunction motif, including a subset of HRGs and genes encoding enzymes involved in the alternative respiratory chain (*AOX1A*, *NDA1, 2*) that allow a oxidative phosphorylation bypass. ANAC017 also regulates *bZIP60* and *bZIP17*, enabling cross-talk between mitochondrial retrograde signaling and the ER. **F)** Mitochondrial ROS also activates MPK3/6 with targets including RAP-type ERFVIIs, which are increased in abundance by higher mitochondrial UCP1 activity. MDM and HRPE (Hypoxia Response Promoter Element) binding sites are recognized by ANACs and ERFVIIs, respectively.

Multiple cellular reactions require molecular O_2_ and therefore have the potential to act as hypoxia sensors, dependent on their respective Michaelis-Menten constant, K_m_O_2_ (van Dongen and Licausi 2015). Beyond the central importance of ERFVII-directed HRG expression, there are ERFVII-independent and ERFVII-intersecting signaling mechanisms that coordinate low O_2_ responses in different subcellular compartments (Holdsworth 2017; Giuntoli et al. 2017b; Zubrycka et al. 2023). Prominent among these is mitochondrial dysfunction. A sudden reduction in O_2_ rapidly attenuates the mETC at Complex IV due to absence of O_2_ as the terminal electron acceptor, leading to a release of at Complex III that triggers mitochondrial retrograde signaling (Chang et al. 2012; Khan et al. 2024) (Fig. 4A). This is partly mitigated by the activity of Uncoupling Protein 1 (UCP1), an abundant inner mitochondrial protein which uncouples ATP synthesis from the proton gradient across the inner membrane to limit ROS production. In parallel, UCP1 upregulation activates HRG transcription by inhibiting the N-degron pathway to link mitochondrial signaling with O_2_ sensing in the cytoplasm (Barreto et al. 2016, 2022). Also, during hypoxia, mitochondria bypass Complex I of the mETC via an alternative respiratory chain comprising Alternative Oxidases (AOXs) and Type II NAD(P)H Dehydrogenases, NDA1 and NDA2 (Jethva et al. 2023). *AOX1a* and *NDA1* are transcriptionally activated by 3 NO APICAL MERISTEM/ARABIDOPSIS TRANSCRIPTION ACTIVATION FACTOR/CUP-SHAPED COTYLEDON (NAC) transcription factors (ANAC013/16/17), which bind to a Mitochondrial Dysfunction Motif (MDM) in their promoters (De Clercq et al. 2013; Ng et al. 2013; Eysholdt-Derzsó et al. 2023) (Fig. 4B). In parallel with the activation of AOX and NDAs, RAP2.2 activates *HRM1*, which in turn attenuates mETC activity and modulates the respiratory chain under hypoxia (Tsai et al. 2023).

Interestingly, pharmacological inhibition of the mETC induces a subset of HRGs, and mitochondrial signaling mutants share common transcriptional signatures with plants subjected to submergence (Wagner et al. 2018; Meng et al. 2020). Underpinning these observations, 31 of the 49 core HRGs of Arabidopsis contain at least 1 copy of the MDM, which enables binding and activation by ANAC013/16/17 (Eysholdt-Derzsó et al. 2023). In the absence of stress, these transcription factors are anchored at the ER membrane through a C-terminal transmembrane domain (Liang et al. 2015). Shortly after imposition of hypoxia stress, ANAC013 is cleaved by Rhomboid-Like Protease 2 (RBL2) and translocates to the nucleus to initiate transcription of HRGs (Eysholdt-Derzsó et al. 2023) (Fig. 4C). Although ANAC013 and ANAC017 positively regulate submergence tolerance, ANAC017 is not released in the initial response to low O_2_, suggesting that it could play a role in prolonged hypoxia/submergence and/or reoxygenation (Bui et al. 2020; Meng et al. 2020; Eysholdt-Derzsó et al. 2023). Consistently, under submergence, ANAC017 regulates genes involved in chloroplast functions and the response to oxidative stress (Ng et al. 2013; Meng et al. 2019). A better understanding of how chloroplasts contribute to hypoxic gene regulation in illuminated photosynthetic organs is needed (Klecker et al. 2014).

Protein folding in the ER is another example of an O_2_-dependent process that integrates hypoxia responses between subcellular compartments. Disulfide bond formation is driven by a relay system in the ER lumen involving protein disulphide isomerases and ER oxidoreductins, with O_2_ as the terminal electron acceptor (Cao et al. 2022; Ugalde et al. 2022) (Fig. 4D). Consequently, both hypoxia and reductive stress impair protein folding and trigger the unfolded protein response (UPR) (Zhou et al. 2021; Fuchs et al. 2022). Moreover, mutants impaired in either ER oxidoreductase activity or the UPR are hypersensitive to hypoxia and reductants such as dithiothreitol (Zhou et al. 2021; Ugalde et al. 2022). The UPR is controlled by 2 distinct signaling pathways. One involves the ER-anchored Basic Leucine Zipper (bZIP) transcription factors bZIP17 and bZIP28, and the other involves ER-localized Inositol-Requiring Enzyme 1 that catalyzes unconventional cytoplasmic splicing of bZIP60, allowing its synthesis and subsequent nuclear localization (Ko and Brandizzi 2024) (Fig. 4D). Intriguingly, the upregulation of *bZIP17* and *bZIP60* by ANAC017 provides a mechanistic link between the UPR and mitochondrial retrograde signaling (Fig. 4, E and F). This serves to protect oxidative protein processing in the ER by boosting mitochondrial respiration (Meng et al. 2019; Fuchs et al. 2022).

### Co- and post-transcriptional response to hypoxia prioritize energy management and prime recovery

**Figure 5.**
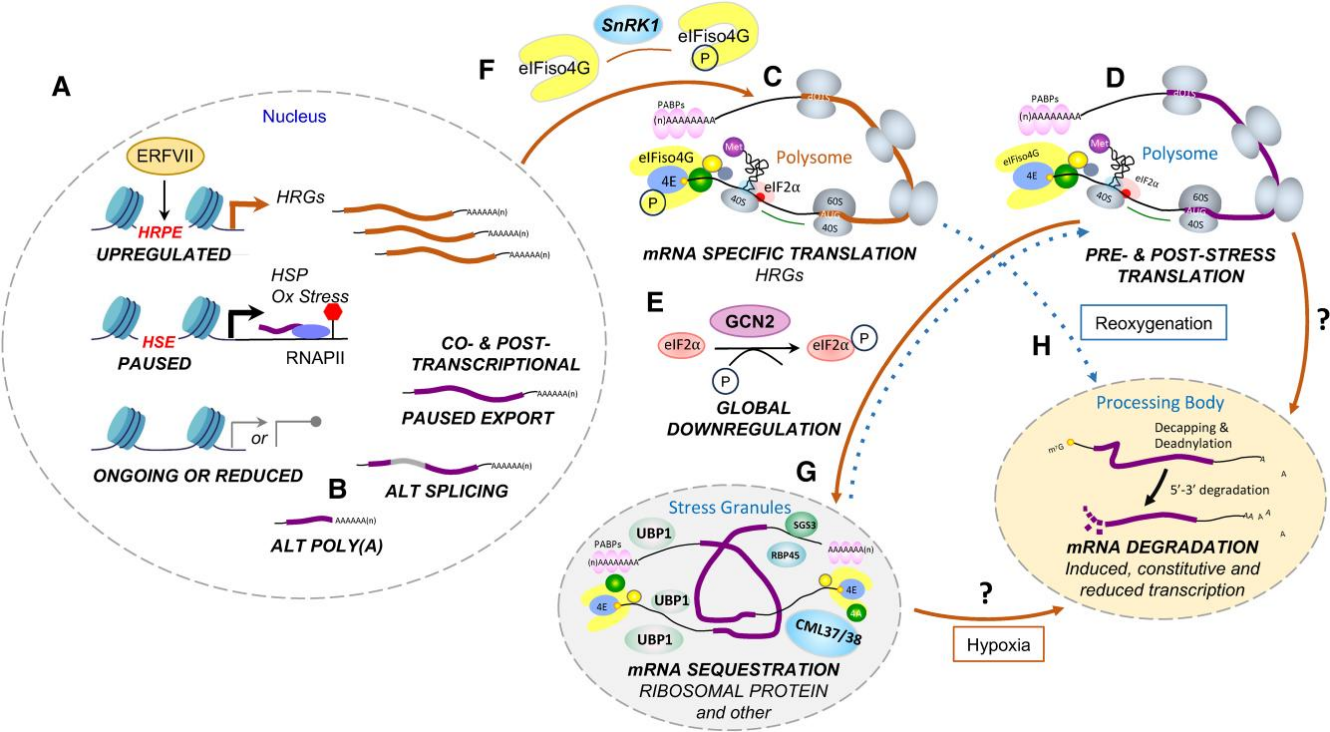
Overview of nuclear and cytoplasmic co- and post-transcriptional regulation in response to hypoxic stress and reoxygenation. **A)** Three scenarios of transcriptional regulation following initial stages of initiation. HRG mRNAs are coordinately synthesized, exported, and translated. Many genes with a Heat Shock Element accumulate high levels of nuclear pre-mRNAs without co-upregulation in total or polysomal mRNA (Lee and Bailey-Serres 2019). These mRNAs either gradually appear in polysomes under hypoxic stress or appear upon reoxygenation. Other genes expressed under aerated conditions can have ongoing or reduced transcription. Genes encoding RIBOSOMAL PROTEINS, for example, continue transcription. **B)** SnRK1 is activated by low cellular sucrose. Among its targets is eIFiso4G upon hypoxia. eIFiso4G-P is used successfully by HRG mRNAs in the initiation phase of translation. eIFiso4G and eIF4G are distinct subunits of the eIFiso4F and eIF4F complexes that recruit 5′-capped and 3′ polyadenylated mRNAs for translation with different specificity. **C)** Hypoxia enhances alternative splicing and polyadenylation site placement. **D)** Translating ribosomes decrease upon hypoxic stress. A global on/off switch for translation is the phosphorylation of eIF2α by the GCN2 kinase, which blocks formation of the ternary complex tRNA-Met-GTP. This complex is an essential component of the pre-initiation complex, comprised of the 40S ribosomal subunit and other proteins. **E)** Upon hypoxia, most mRNAs dissociate from polysomes, and those that are stable associate with the constitutively present RNA binding protein UBP1C until this is reversed by reoxygenation. UBP1C and the hypoxia-induced RNA binding protein CML38 form heterogenous biomolecular condensates in the cytoplasm containing RNA and other proteins. UBP1C and CML38 may form interacting or distinct complexes. **F)** Little is known about the impact of hypoxia and reoxygenation on mRNA degradation pathways. Likely important are the heterogenous cytoplasmic condensates called processing bodies that organize the removal of the protective 5′ ^m7^G cap and 3′ polyA tail of mRNAs (Chantarachot and Bailey-Serres 2018). Orange lines, hypoxia; blue dotted lines, reoxygenation.

Hypoxia has a pronounced effect on gene regulation following transcriptional initiation (Fig. 5, A to H). In Arabidopsis, hypoxia-modulated co-transcriptional processes include pausing of RNAPII, alternative splicing, and alternative polyadenylation site selection (Juntawong et al. 2014; de Lorenzo et al. 2017; Lee and Bailey-Serres 2019). Transient pausing of RNAPII is prevalent on genes associated with heat and oxidative stress and, depending on the gene, is released during the stress or upon reaeration (Lee and Bailey-Serres 2019). This suggests certain RNAPII complexes are engaged but require a signal to be released, such as the ROS burst upon reoxygenation. A comparison of transcripts with similar abundance under control and hypoxic conditions identified extensive intron retention in mRNAs encoding splicing factors, as well as alternative splicing in induced and reduced transcripts (Juntawong et al. 2014). Alternative splicing of the HRG *HRE1*, an ERFVII, results in synthesis of 2 protein isoforms shown to differ in transactivation activity in protoplasts (Seok et al. 2020). Hypoxia also alters the site of polyA tail addition on some mRNAs, resulting in truncated or lengthened mRNAs (de Lorenzo et al. 2017) (Fig. 5B). Premature polyadenylation in introns of *NITRATE REDUCTASE1 and 2* results in transcripts encoding truncated enzymes that retain the active site for nitrite production (de Lorenzo et al. 2017). These isoforms may bolster nitrite levels to augment NO production upon reoxygenation to promote ERFVII turnover. Altered polyA site selection could be symptomatic of reduced RNAP II processivity, alterations in the polyadenylation apparatus, or N^6^-methyadenosinene modification of specific adenosines of transcripts. N^6^-methyadenosinene is bound by readers that interact with other proteins to direct mRNA polyadenylation, splicing, turnover, and translation. This is yet to be studied in the context of hypoxia in plants.

Although polyA^+^ mRNA is routinely used to monitor gene activity, other subpopulations of transcripts can be isolated and profiled, including nuclear, ribosome associated (translatome), or RNA-binding protein associated (Lee and Bailey-Serres 2017). In fact, the Arabidopsis HRGs were recognized as the set of 49 gene transcripts with increased translation across cell types of roots and shoots in hypoxic seedings (Mustroph et al. 2009). In addition, each cell type has a distinct pattern of differentially translated mRNAs under hypoxia; for example, certain sucrose transporters are preferentially upregulated in the root phloem. Perhaps ERFVIIs or other transcription factors that regulate the core hypoxia response also target genes that are regulated in a cell-specific manner. Precise mapping of individual ribosomes on mRNAs confirmed that *HRG* mRNAs are highly translated during hypoxia (Fig. 5C), whereas mRNAs encoding many proteins, including those needed to build cytosolic ribosomes, are stable but disassociate from ribosomes (Branco-Price et al. 2008; Juntawong et al. 2014) (Fig. 5D). Submergence also invokes preferential translation of HRG mRNAs in seedlings of Arabidopsis (Cho et al. 2022) and of conserved submergence-upregulated mRNAs, including HRGs in root tips of rice, tomato, and medicago (Reynoso et al. 2019). The coordinated decline in translating ribosomes and ATP during hypoxia and recovery upon reoxygenation supports the hypothesis that translation, which is highly ATP demanding, is globally repressed under hypoxia as a general energy management strategy (Branco-Price et al. 2008). But how might this global and mRNA-specific translation be orchestrated?

Translation is intertwined with processes of mRNA turnover and sequestration (Browning and Bailey-Serres 2015; Chantarachot and Bailey-Serres 2018). GENERAL CONTROL NONDEREPRESSIBLE (GCN2) controls overall levels of translation by limiting formation of the eukaryotic initiation factor 2α (eIF2α)-tRNA-Met complex required to complete the initiation phase (Cho et al. 2022). GCN2 phosphorylates eIF2α via an ethylene-activated pathway within an hour of seedling submergence, reducing overall translation (Fig. 5E). Remarkably, this eIF2α phosphorylation facilitates translation of tested HRGs under the stress. Translational control at the mRNA-specific level, on the other hand, can involve the energy sensing Snf1-related protein kinase (SnRK1). SnRK1 activation within 30 min of submergence triggers phosphorylation of eukaryotic Initiation Factor (eIF)iso4G1 (Cho et al. 2019) (Fig. 5F). This protein is important in preparing mRNA for scanning by a pre-initiation complex carrying eIF2α-tRNA-Met (Browning and Bailey-Serres 2015), and SnRK1-phosphorylated eIFiso4G fosters translation of certain HRGs. A determinant of this may be an unstructured 5′ UTR, presumed to require less ATP for translational initiation. Another example of mRNA-specific translational regulation under hypoxia is observed for S1 class bZIP transcription factor mRNAs. The 5′ UTRs of these possess a conserved polypeptide-encoding upstream open reading frame that causes ribosomes to stall before reaching the ORF encoding the bZIP, in a sucrose-dependent manner. Ribosome footprinting studies revealed that gatekeeping by the conserved polypeptide-encoding upstream open reading frame of these mRNAs is derepressed by hypoxia (Juntawong et al. 2014).

Intriguingly, mRNAs that are poorly translated during hypoxia associate with the RNA binding protein OLIGOURIDYLATE BINDING PROTEIN 1C (UBP1C) that assembles into cytoplasmic condensates within minutes of hypoxia and rapidly dissipates upon reoxygenation (Sorenson and Bailey-Serres 2014) (Fig. 5G). A comparison of ribosome-associated and UBP1C-associated mRNAs confirmed a rapid shift in mRNAs that encode ribosomal protein from polyribosomes to UBP1C complexes under hypoxia. Upon reoxygenation, these and other UBP1C-bound mRNAs rapidly reassociate with ribosomes. Other RNA binding proteins are important during hypoxic stress. CALMODULIN LIKE38 (CML38), encoded by a *HRG* essential for resilience, also forms cytoplasmic condensates during hypoxia. These contain proteins associated with mRNA sequestration (RBP47B) and mRNA silencing (SUPPRESSOR OF SILENCING 3) (Lokdarshi et al. 2016; Field et al. 2021). The calmodulin domain of CML38 could integrate cytosolic Ca^2+^ dynamics with mRNA control under hypoxia. Thus, the ability of plants to prioritize the translation of *HRG* mRNAs over other mRNAs during hypoxia may reflect their ability to circumvent sequestration. This targeted curtailment of mRNA translation, perhaps because of GCN2 activation, limits ATP consumption and protects many mRNAs from decay until reoxygenation (Fig. 5H). There is still much to be learned about RNA regulation in the context of hypoxia.

## Concluding remarks

The processes of gene regulation in response to hypoxic stress—from chromatin through translation and the associated diversity of post-translational processes—involve complex and intertwined mechanisms. We have highlighted the importance of changes in O_2_ availability, ethylene, energy, and second messengers such as Ca^2+^, as well as crosstalk across organelles and a complex network of post-translational protein modifications. Despite the breadth of knowledge gained using Arabidopsis, there are many **Outstanding Questions**. Moreover, there is little knowledge of the conservation of hypoxia-response mechanisms within and across species. Given what has been gleaned by within-species comparisons, such as ERFVII variation at high altitudes (Abbas et al. 2022) and variation gene regulatory circuitry in floodland- vs dryland- adapted species (Reynoso et al. 2019), there is likely much to be learned from natural variation.

**OUTSTANDING QUESTIONS**

- What controls hypoxia response signatures in cell types and tissues across the life cycle?
- How is the interplay between N-degron-ERFVII, ER stress, mitochondrial, and other retrograde signaling modules and genes regulated? Do these connect to cell-specific networks controlling metabolism, growth, and development?
- Do plants encode bona fide hypoxia stress “memories” at the chromatin level, and under what timescales are these initiated and maintained?
- How is the response silenced at the protein level as O_2_ levels recover? Are Ca^2+^, ROS, redox state, and ATP levels involved?
- Is transcription primed for reoxygenation? Do hypoxia-responsive or reoxygenation-triggered phosphatases counterbalance phosphorylation of ERFVII by MPKs and CPKs?
- What determines which mRNAs are translated, sequestered into condensates, or degraded during hypoxia and upon reaeration?

## Data Availability

No new data were generated or analysed as part of this update article.

## Dive Curated Terms

The following phenotypic, genotypic, and functional terms are of significance to the work described in this paper:

ACBP4 Gramene: AT3G05420
ACBP4 Araport: AT3G05420

NDA1 Gramene: AT1G07180
NDA1 Araport: AT1G07180

AGO1 Gramene: AT1G48410
AGO1 Araport: AT1G48410

ACBP1 Gramene: AT5G53470
ACBP1 Araport: AT5G53470

PIF4 Gramene: AT2G43010
PIF4 Araport: AT2G43010

FLC Gramene: AT5G10140
FLC Araport: AT5G10140

HR4 Gramene: AT3G50480
HR4 Araport: AT3G50480

BZIP17 Gramene: AT2G40950
BZIP17 Araport: AT2G40950

AOX1A Gramene: AT3G22370
AOX1A Araport: AT3G22370

SR1 Gramene: AT1G02840
SR1 Araport: AT1G02840

NDA2 Gramene: XP_001703643
NDA2 Araport: XP_001703643

ZPR2 Gramene: AT3G60890
ZPR2 Araport: AT3G60890

EIN2 Gramene: AT5G03280
EIN2 Araport: AT5G03280

VRN2 Gramene: At4g16845
VRN2 Araport: At4g16845

VIN3 Gramene: AT5G57380
VIN3 Araport: AT5G57380

HRE2 Gramene: AT2G47520
HRE2 Araport: AT2G47520

UCP1 Gramene: AT3G54110
UCP1 Araport: AT3G54110

NADPH CHEBI: CHEBI:16474

h3k27me3 CHEBI: CHEBI:85045

bZIP60 Gramene: At1g42990
bZIP60 Araport: At1g42990
